# Assessment of National Public Health and Reference Laboratory, Accra, Ghana, within Framework of Global Health Security

**DOI:** 10.3201/eid2313.170372

**Published:** 2017-12

**Authors:** Adaeze Ogee-Nwankwo, David Opare, Gifty Boateng, Mawuli Nyaku, Lia M. Haynes, S. Arunmozhi Balajee, Laura Conklin, Joseph P. Icenogle, Paul A. Rota, Diane Waku-Kouomou

**Affiliations:** Centers for Disease Control and Prevention, Atlanta, Georgia, USA (A. Ogee-Nwankwo, M. Nyaku, L.M. Haynes, S.A. Balajee, L. Conklin, J.P. Icenogle, P.A. Rota);; National Public Health and Reference Laboratory, Accra, Ghana (D. Opare, G. Boateng);; IHRC, Inc., Atlanta (D. Waku-Kouomou)

**Keywords:** global health security, measles, rubella, measles virus, rubella virus, viruses, public health, reference laboratory, assessment, Accra, Ghana

## Abstract

The Second Year of Life project of the Global Health Security Agenda aims to improve immunization systems and strengthen measles and rubella surveillance, including building laboratory capacity. A new laboratory assessment tool was developed by the Centers for Disease Control and Prevention to assess the national laboratory in Ghana to improve molecular surveillance for measles and rubella. Results for the tool showed that the laboratory is well organized, has a good capacity for handling specimens, has a good biosafety system, and is proficient for diagnosis of measles and rubella by serologic analysis. However, there was little knowledge about molecular biology and virology activities (i.e., virus isolation on tissue culture was not available). Recommendations included training of technical personnel for molecular techniques and advocacy for funding for laboratory equipment, reagents, and supplies.

The International Health Regulations (*1*) recommends that countries develop, strengthen, and maintain the capacity to detect, notify, and report major events resulting in public health risk and emergencies of international concern, such as infectious disease epidemics. The difficulties encountered in providing timely laboratory testing during the recent epidemic of Ebola in West Africa (*2*) highlighted that global health security relies on adequate public health laboratory capacity in all countries, including Ghana. The 2012–2020 Global Measles and Rubella Strategic Plan calls for effective case-based surveillance of measles and rubella with laboratory confirmation (*3*).

The World Health Organization (WHO) recommends that all countries implement virologic surveillance of measles and rubella to help identify sources of infection and verify elimination (*4*). The WHO Global Measles and Rubella Laboratory Network (GMRLN), established in 2000, has >700 laboratories serving 191 countries, providing diagnostic support for measles and rubella surveillance (*5*). As of 2015, only 48% of countries reporting laboratory-confirmed measles cases also reported measles virus genotypes, and only 10% of countries reporting laboratory-confirmed rubella cases also reported rubella virus genotypes (*6*).

To support the WHO/GMRLN recommendations for measles and rubella surveillance, including virologic surveillance, the Measles and Rubella Global Specialized Laboratory (GSL) (Division of Viral Diseases, National Center for Immunization and Respiratory Diseases) at the Centers for Diseases Control and Prevention (CDC, Atlanta, GA, USA) supports laboratory capacity building in all WHO regions. The global reach of the GSL at CDC enabled partnering with the Global Health Security Agenda (GHSA), launched in 2014 and aimed at prevention, detection, and response to infectious diseases outbreaks worldwide (*7*).

Laboratories play a critical role in the surveillance of measles and rubella, which requires high-quality testing. However, there is currently no tool to assess the capacity of a laboratory, especially for measles and rubella surveillance or to compare different laboratories within the GMRLN. In response to the need for a standardized capacity measurement tool, the CDC GSL developed the CDC International Measles and Rubella Laboratory Capacity Review tool. This tool was field tested at the National Public Health and Reference Laboratory (NPHRL) in Accra, Ghana, as part of the Second Year of Life Project, within the GHSA. This project aims to improve immunization systems and to strengthen disease surveillance for vaccine-preventable disease, including building laboratory capacity for surveillance of measles and rubella and supporting implementation of surveillance for congenital rubella syndrome.

In Ghana, the NPHRL, which is a GMRLN laboratory, currently performs testing to detect measles- or rubella-specific IgM. The capacity to conduct molecular testing is minimal. The objectives of the assessment of the capacity of NPHRL were to describe the status of the laboratory and determine the needs for equipment and training required to initiate molecular testing. We describe the new CDC International Measles and Rubella Laboratory Capacity Review tool and the results of the laboratory capacity assessment of the NPHRL.

The CDC International Measles and Rubella Laboratory Capacity Review tool was created in Excel (Microsoft, Redmond, WA, USA) by using the International Influenza laboratory capacity tool (*8*,*9*) as a model. The tool is organized into 8 sections. Each section is composed of a set of questions that guide the process of assessing laboratory capacity to help identify the strengths and challenges of the laboratory, including priority areas for strengthening: 1) general laboratory (39 questions), 2) specimen collection and reporting (32 questions), 3) virology laboratory (19 questions), 4) molecular biology (27 questions), 5) laboratory biosafety and safety (31 questions), 6) quality assurance/quality control (20 questions), 7) equipment (11 questions), and 8) training (36 questions). These questions aimed to identify the capacity of a laboratory to frequently respond to public health events, such as a measles and rubella outbreak, by accurately testing specimen and reporting data in a timely manner; identify safety and biosafety measure implementation in place; and professional development of laboratory staff. These questions also helped to collect information on the role of the laboratory in public health surveillance; and conditions of the facility, including the building, availability of electricity, water, and air conditioning.

Each question was assigned a point value of 1 or 0, except for multiple option questions, for which each option was assigned either a value of 0.25 or 0.5 to minimize total score difference between questions in the same section. Weighting of questions was not applied because the tool was used to capture areas of strength and weaknesses to enable the country to prioritize areas that need to be strengthened first on the basis of their public health objectives and available resources.

Assessment data were entered into an Excel-based file and scores were calculated. The points for each section were automatically summed and divided by the total number of points available in the section and converted into a percentage. The assessment of NPHRL was conducted during 5 days in March 2016 by 2 subject matter experts from CDC who had expertise in laboratory methods, laboratory capacity building, and surveillance for measles and rubella. These experts conducted a site visit to NPHRL to interview laboratory personnel, evaluate facilities, and review key documents. Two laboratory assessment tools were used to capture information on public health functions. The first tool used was the WHO Laboratory Assessment Tool (*10*), which broadly captures all aspects of laboratory services. The second tool used was the new CDC International Measles and Rubella Laboratory Review Tool, which focuses on measles and rubella–specific laboratory testing activities, such as virus isolation, confirmation of measles and rubella infection, and genotyping of measles and rubella viruses.

Results obtained with the WHO tool indicated that NPHRL is well organized and has a functioning quality management system (Figure 1). However, equipment, reagents, and supplies are usually insufficient, mostly because of a lack of funding coupled with unavailability of reagents in the country. Some critical reagents and supplies have to be ordered from outside Ghana, and this factor results in delay. Major challenges include inadequate financial resources for laboratory activities and maintenance of equipment and lack of political commitment (e.g., policies, budget) to support the laboratory (Figure 2).

**Figure 1:**
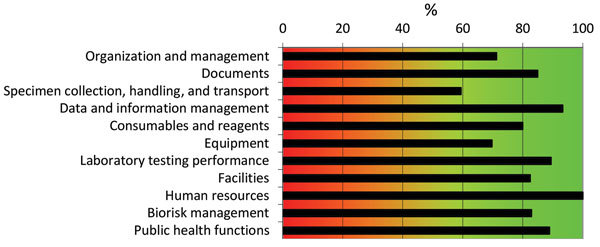
Summary of assessment results for the National Public Health and Reference Laboratory, Accra, Ghana, determined by using the World Health Organization Laboratory Assessment Tool. Capacity score (0%–100%) of each section of the tool is indicated and color coded. Red (<50%) indicates need for major improvement; orange (50%−80%), some improvement is necessary; green (>80%), the laboratory is in good standing.

**Figure 2:**
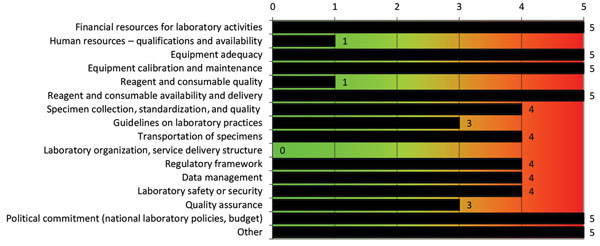
Gap score analysis of the National Public Health and Reference Laboratory, Accra, Ghana, performed by using the World Health Organization Laboratory Assessment Tool. Gaps are indicated on the basis of a score of 0–5. Results are indicated with a color code for each section of the laboratory. Green (0–1), no gaps found; orange (2,3), needs some improvement; red (4,5), requires major improvement. Other, lack of political commitment.

Results obtained with the CDC tool showed good capacity for specimen handling (100%) and biosafety and safety (81%) (Figure 3). However, there was little capacity for virology (0%) or molecular biology (2%) (Figure 3). The NPHRL was proficient in serologic testing for measles and rubella because this laboratory passed its 2015 ELISA proficiency test as part of the GMRLN proficiency testing program coordinated by WHO. None of the NPHRL staff assigned to measles and rubella serologic testing was trained in molecular biology techniques for measles and rubella surveillance.

**Figure 3:**
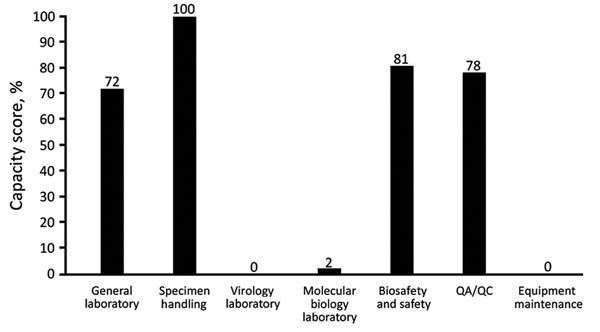
Indicators of laboratory capacity for measles and rubella at the National Public Health and Reference Laboratory, Accra, Ghana, analyzed by using the Centers for Disease Control and Prevention International Measles and Rubella Laboratory Review Tool. Capacity score is indicated (0%–100%) for each section in the tool. QA, quality assurance; QC, quality control.

Since 2012, the NPHRL has been involved in the Strengthening Laboratory Management Toward Accreditation (SLMTA) program (*11*). The SLMTA scored checklist quantifies the quality status of a laboratory by using a 0–5-star rating (*12*). The NPHRL has received 1 SLMTA star since December 2013. Overall, NPHRL had a score of 72% by the CDC tool (Figure 3) and a score of 71% by the WHO tool (Figure 1). Furthermore, both tools confirmed weakness in maintenance of laboratory equipment and showed the highest gap score (5) by the WHO tool (Figure 2) and the lowest capacity score by the CDC tool (0%) (Figure 3). Gap score analysis with the WHO tool (Figure 2) resulted from a set of questions asked to laboratory staff to highlight and prioritize the biggest needs or weaknesses of the laboratory. Thus, gap scores might be interdependent and not directly proportional to the capacity score observed (Figure 1).

For NPHRL, lack of financial resources, which had the highest gap score (5), directly affected the possibility of performing regular calibration and maintenance of equipment and the availability of equipment, reagents, and consumables (Figure 2). In addition, lack of political commitment made it difficult to maintain the facilities (shown as “other” in Figure 2). Specimen collection, which had the lowest score (59%) (Figure 1), was classified as a second priority, with a gap score of 4 (Figure 2).

The main advantage of the CDC tool is its specificity in regards to measles and rubella laboratory activities. Therefore, recommendations based on assessment results covered all requirements needed to strengthen measles and rubella laboratory surveillance. This new tool could also be quickly adapted to assess laboratory activities for surveillance of other viral diseases.

This study had some limitations. Both tools did not capture the same information. Therefore, it is difficult to fully compare these tools. The CDC tool does not capture laboratory testing activities for diseases other than measles and rubella, whereas the WHO tool captures these laboratory activities. Thus, there were some discrepancies observed between results obtained with the WHO tool compared with those obtained with the CDC tool regarding specimen handling, for which the scores were 60% and 100%, respectively. Such a difference was also found in laboratory testing performance, for which the score was 80% with the WHO tool (Figure 1) compared with 0%–2% (virology laboratory and molecular biology laboratory) with the CDC tool (Figure 3).

The CDC tool was critical in capturing laboratory-specific activities needed for measles and rubella surveillance and to rapidly identify related laboratory needs, such as specific equipment required for molecular and virologic testing, training of laboratory personnel for molecular methods for case confirmation and genotyping, and the need for training for tissue culture and virus isolation. The CDC and WHO tools complemented each other in providing a more complete picture of the capacity of NPHRL. For example, the WHO tool provided information on human resources, consumables, and reagents, as well as public health functions of the NPHRL. The CDC tool focused on information related to laboratory activities, such as virology and molecular biology for measles and rubella surveillance.

The assessment results were used to develop a working plan for improving molecular surveillance of measles and rubella in Ghana, which is needed to support achievement of the 2020 measles elimination goal. Laboratory activities will focus on implementation of molecular methods for case confirmation and genetic characterization of measles and rubella virus strains. Equipment and reagent needs will be supported, and laboratory personnel will be trained by the end of 2017, with support from the GHSA and CDC GSL. The data produced from this set of activities will be sent to the Ghana Ministry of Health, the WHO country office, and the WHO Regional Laboratory Coordinator for the West African Region. These data can be used to advocate for more financial resources from the Ghana Ministry of Health, WHO, and other partners to ensure the sustainability of laboratory surveillance of measles and rubella at NPHRL.

Continual reassessment by using the same tools will help to measure the effect of GHSA support at NPHL. The new CDC tool (which is available upon request to the corresponding author) will also be used to assess measles and rubella laboratories in other countries within the GMRLN as needed by WHO, and could be adapted to assess laboratory capacity for other vaccine-preventable diseases worldwide. Building laboratory capacity and especially building molecular biology capacity for measles and rubella surveillance will strengthen the NPHRL platform for detection of other diseases and increase the capacity of a country to rapidly detect, respond, and contain public health emergencies at their source, thereby enhancing global health security.
